# Metastasis risk prediction model in osteosarcoma using metabolic imaging phenotypes: A multivariable radiomics model

**DOI:** 10.1371/journal.pone.0225242

**Published:** 2019-11-25

**Authors:** Heesoon Sheen, Wook Kim, Byung Hyun Byun, Chang-Bae Kong, Won Seok Song, Wan Hyeong Cho, Ilhan Lim, Sang Moo Lim, Sang-Keun Woo

**Affiliations:** 1 Division of applied RI, Korea Institute of Radiological and Medical Sciences (KIRAMS), Seoul, Republic of Korea; 2 Departments of Nuclear Medicine, Korea Institute of Radiological and Medical Sciences (KIRAMS), Seoul, Republic of Korea; 3 Department of Orthopedic Surgery, Korea Institute of Radiology and Medical Sciences, Seoul, Republic of Korea; Ente Ospedaliero Cantonale, SWITZERLAND

## Abstract

**Background:**

Osteosarcoma (OS) is the most common primary bone tumor affecting humans and it has extreme heterogeneity. Despite modern therapy, it recurs in approximately 30–40% of patients initially diagnosed with no metastatic disease, with the long-term survival rates of patients with recurrent OS being generally 20%. Thus, early prediction of metastases in OS management plans is crucial for better-adapted treatments and survival rates. In this study, a radiomics model for metastasis risk prediction in OS was developed and evaluated using metabolic imaging phenotypes.

**Methods and findings:**

The subjects were eighty-three patients with OS, and all were treated with surgery and chemotherapy for local control. All patients underwent a pretreatment ^18^F-FDG-PET scan. Forty-five features were extracted from the tumor region. The incorporation of features into multivariable models was performed using logistic regression. The multivariable modeling strategy involved cross validation in the following four steps leading to final prediction model construction: (1) feature set reduction and selection; (2) model coefficients computation through train and validation processing; and (3) prediction performance estimation. The multivariable logistic regression model was developed using two radiomics features, SUVmax and GLZLM-SZLGE. The trained and validated multivariable logistic model based on probability of endpoint (P) = 1/ (1+exp (-Z)) was Z = -1.23 + 1.53*SUVmax + 1.68*GLZLM-SZLGE with significant p-values (SUVmax: 0.0462 and GLZLM_SZLGE: 0.0154). The final multivariable logistic model achieved an area under the curve (AUC) receiver operating characteristics (ROC) curve of 0.80, a sensitivity of 0.66, and a specificity of 0.88 in cross validation.

**Conclusions:**

The SUVmax and GLZLM-SZLGE from metabolic imaging phenotypes are independent predictors of metastasis risk assessment. They show the association between ^18^F-FDG-PET and metastatic colonization knowledge. The multivariable model developed using them could improve patient outcomes by allowing aggressive treatment in patients with high metastasis risk.

## Introduction

Osteosarcoma (OS), the most common primary bone tumor affecting humans, develops in children and adolescents between the ages of ten and twenty-five years, and in persons with deficient bone remodeling [1,2]. The survival rate of these patients has significantly improved as a result of comprehensive management in the form of intensive chemotherapy and surgery [1,3]. However, despite modern therapy, OS recurs in approximately 30–40% of patients initially diagnosed with no metastatic disease [4,5]. The long-term survival rates of such patients is generally 20% [6,7]. Consequently, individual and early prediction of metastases in the plan of OS management is crucial as it could result in better-adapted treatments and survival rate.

Currently, the most important predictors are tumor grade, size, and presence of skip lesions or distant metastases, which are found from biopsies and microscopic examinations [1,8]. However, they do not always provide sufficient results because the risk of relapse may differ among patients with the same disease stage or chemotherapeutic response owing to the heterogeneity of the tumor population [4,8,9]. Within this context, imaging modalities and, more particularly, ^18^F-FDG positron emission tomography (PET) are generally used for staging and monitoring various cancers. As its accumulation explains the characteristics of heterogeneity in tumors, it is increasingly being accepted as the prognostic value [10,11]. Moreover, the analysis of radiomics features from ^18^F-FDG PET imaging has recently been considered as additional information for predicting tumor response to therapy [12] because it could theoretically provide further understanding of the hidden tumor biology as compared with maximum standard uptake value (SUVmax), mean SUV (SUV_mean_), peak SUV (SUV_peak_), metabolic tumor volume (MTV), and total lesion glycolysis (TLG).

The advent of personalized medicine has increased the need for the improvement of clinically feasible prediction models for treatment decision [13]. After radiomics features from ^18^F-FDG PET imaging are decided by the relation to a given tumor outcome as prognostic factors, models combining those factors need to be developed in order to make better prediction performance. Multivariable models could further fully characterize intratumoral heterogeneity, even though univariate analysis has been deemed informative. Based on the above considerations, this study was conducted with the objective of developing a multivariable model based on optimal radiomics features from pretreatment ^18^F-FDG PET imaging in order to assess metastasis risk at initial diagnosis. To achieve this main objective, firstly, the relevant features were selected from a large number of features by the statistical methods for radiomics model. Secondly, multivariable modeling strategies were formulated to develop texture-based models with optimal predictive and generalized properties. Finally, the final optimal model was evaluated using the test dataset. This developed radiomics model and the selected significant features are expected to ultimately encourage physicians to make better decisions for treatment and potentially improve survival rate.

## Materials and methods

### Patient cohort

A database of eighty-three consecutive patients was retrospectively retrieved between June 2006 and August 2010. The patient qualification requirements were as follows: newly diagnosed, histologically biopsy-proven primary intramedullary osteosarcoma; completion of neoadjuvant chemotherapy and surgery; less than two weeks between ^18^F-FDG PET/CT scan and initiation of preoperative chemotherapy; and no history of previous treatment except for biopsy. Patients with metastatic osteosarcoma at the present time were excluded from the study. Metastases were either proven by biopsy or diagnosed by an expert physician from the following-up cross-sectional imaging results of ^18^F-FDG PET/CT imaging, MR imaging, bone scanning, and x-ray for at least six months. Informed consent was waived by the ethics committee because of the retrospective nature of this study and the analysis using anonymous imaging and clinical data. This study was approved by the Institutional Review Board of KIRAMS (IRB No.: K-1707-001-004 (eIRB NO.: 2017-04-004)). Patient characteristics and histologic features are described in Table 1.

**Table 1:** Patient characteristics.

Characteristics	Value
Sex, n (%)	
Female	23 (27.71%)
Male	60 (72.29%)
Age, n (%)	
years ≤ 19	67 (80.72%)
years >19	16 (19.27%)
Location of primary tumor, n (%)	
Humerus	4 (4.82%)
Radius	2 (2.41%)
Femur	44 (53.01%)
Fibula	3 (3.61%)
Tibia	30 (36.14%)
AJCC stage, n (%)	
IIA	35 (42.2%)
IIB	48 (57.8%)
Pathologic subtype, n (%)	
OB (Osteoblastic)	60 (72.29%)
CB (Chorndroblastic)	9 (10.84%)
FB (Fibroblastic)	7 (8.43%)
Others	7 (8.43%)

Good historic response was 46.99% of the patients and poor historic response was 53.01% of the patients in neoadjuvant chemotherapy outcomes. Of the patients, 26.51% had metastasis (lung metastasis: 72.22%, bone metastasis: 27.78%) after completion of neoadjuvant chemotherapy and surgery in five years or seven years (one patient). The remaining 73.49% of patients were metastasis-free until a composite endpoint determined by the time from the date of diagnosis to the date of metastasis (Table 2).

**Table 2:** Characteristics of clinical outcomes.

Clinical outcomes	Total Number (n)	83 (100%)
Histologic response	Good	39 (46.99%)
Metastasis	Poor	44 (53.01%)
	No	61 (73.49%)
	Yes	22 (26.51%)
Histologic response and Metastasis	Good-MetaFree (Good Response and Metastasis Fress)	34 (40.96%)
	Poor-MetaFree (Poor Response and Metastasis Free)	27 (32.53%)
	Good-Meta (Good Response and Metastasis)	5 (6.02%)
	Poor-Meta (Poor Response and Metastasis)	17 (20.48%)

^18^F-FDG PET/CT Imaging Data

All eighty-three qualified patients had performed pre-treatment ^18^F-FDG PET/CT scans using a Biography6 PET/CT scanner (Siemens Medical Solutions, Erlangen, Germany) at Korean Cancer Center Hospital (KCCH). All patients were instructed to fast for at least six hours before the intravenous administration, with water intake permitted and encouraged. Prior to administration of ^18^F-FDG (injected dose: 7.4 MBq per kg of body weight), a blood glucose level of <7.2 mmol/L was confirmed. The CT scan (130 kVp, 30 mA, 0.6 sec/rotation, pitch 6) without contrast agent and 3D PET scan (16.2 cm axial field of view, 3.5 min/bed position) were obtained for the sites of tumors located in the extremities (the vertex to the upper thigh). PET images were reconstructed using the ordered-subsets expectation maximization (OSEM) algorithm (two iterations and eight subsets) with CT-based attenuation correction after normalization, correction for scatter, random, decay, and dead time, and smoothed using a 5 mm post Gaussian filter to control noise. The ^18^F-FDG PET slice thickness was 3.03 mm and its matrix size was 4.063 mm × 4.063 mm.

### Tumor volume definition and features extraction

Tumor volumes were segmented and radiomics features in the defined tumors subsequently extracted using the Local Image Features Extraction (LIFEx) version 4.0 software package (http://www.lifexsoft.org) [14–16]. The tumor region was drawn using a semi-automated segmentation method with threshold SUV of 2.0 based on our previous report [9] in three-dimensional (3D) images. In the segmented tumors, SUVmax, SUVmean, SUVpeak, Metabolic Tumor Volume (MTV), and Total Lesion Glycolysis (TLG) and features from shape and histogram were calculated as the first-order features.

For texture feature calculation, the numbers of intensity levels were resampled using 64 discrete values between zero and 20 SUVs, corresponding to a sampling bin width of 0.3125 SUVs [14,16,17]. Spatial resampling was 4.1 mm (X-direction), 4.1 mm (Y-direction), and 2.5 mm (Z-direction) in Cartesian coordinates [14].

Texture features were assessed by four texture matrices: the co-occurrence matrix (CM), the gray-level run length matrix (GRLM), the gray-level zone length matrix (GZLM), and the neighborhood gray-level different matrix (NGLDM). The CM was calculated in 13 directions with one voxel distance relationship between neighboring voxels, and each texture feature calculated from this matrix was the average of the features over the 13 directions in space (X, Y, Z). The GRLM was also calculated for 13 directions via a similar method while the GZLM was computed directly in 3D. NGLDM was computed from the difference of gray-levels between one voxel and its 26 neighbors in 3D and each texture feature was calculated from this matrix.

Forty-five features were extracted from the analysis of the volumes inspected: five conventional features, five histogram features, three shape features, and thirty-one texture features (Table 3).

**Table 3:** Summary of the first-order, second-order, and high-order features index.

Order of extracted feature	Matrix	Index	Type
First order	Conventional features	SUVmin (minimum SUV) SUVmax SUVpeak SUVmean TLG	Global
	Histogram features	Skewness Kurtosis Entropy_log10 Entropy_log2 Energy	
	Shape features	Sphericity Compacity Volume (MTV)	
Second order	GLCM (Gray-Level Co-occurrence based on concurrence Matrix)	Homogeneity Energy Correlation Contrast Entropy Dissimilarity	Regional

	GLRLM (Gray-Level Run Length based on voxel-alignment Matrix)	SRE (short-run emphasis) LRE (long-run emphasis) LGRE (low grey-level run emphasis) HGRE (high grey-level run emphasis) SRLGE (short-run low grey-level emphasis) SRHGE (short-run high grey-level emphasis) LRLGE (long-run low grey-level) LRHGE (long-run high grey-level emphasis) GLNUr (grey-level non-uniformity for run) RLNU (run-length non-uniformity) RP (run percentage)	Regional

High order	NGLDM (Neighborhood Gray-Level Different based on neighborhood intensity-difference Matrix)	Coarseness Contrast Busyness	Local
	GLZLM (Gray-Level Zone Length based on intensity–size–zone Matrix)	SZE (short-zone emphasis) LZE (long-zone emphasis) LGZE (low grey-level zone emphasis) HGZE (high grey-level zone emphasis) SZLGE (short-zone low grey-level emphasis) SZHGE (short-zone high grey-level emphasis) LZLGE (long-zone low grey-level emphasis) LZHGE (long-zone high grey-level emphasis) GLNUz (grey-level nonuniformity for zone) ZLNU (zone length non-uniformity) ZP (zone percentage)	Local


### Feature selection and evaluations

**Fig 1:**
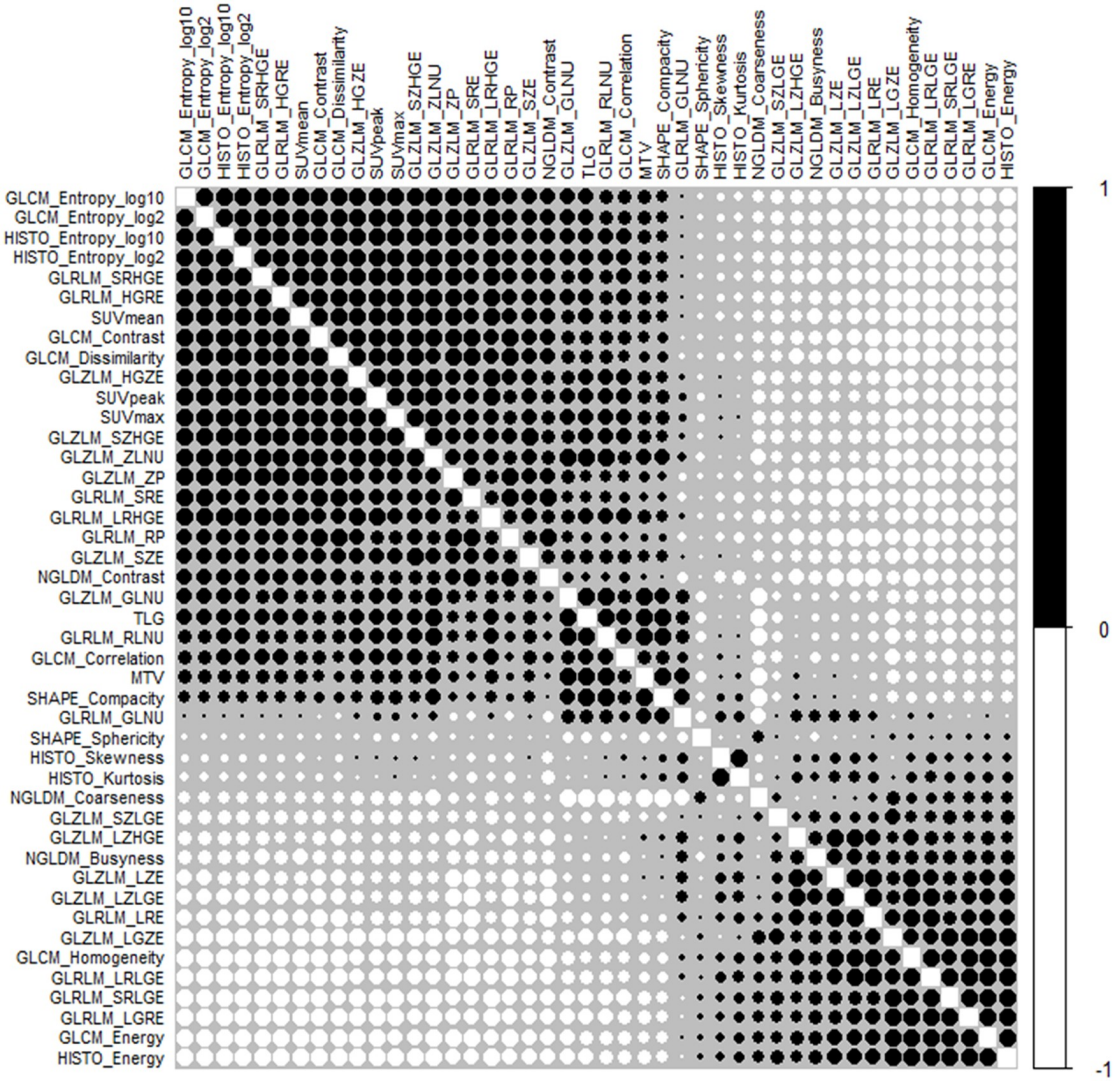
Spearman rank correlation of radiomics features in the training dataset. Forty-five features were extracted from tumor volumes in eighty-three patients. Across all tumors, the correlation of each feature with all other features was investigated via Spearman’s rank correlation. The color and size of the circle indicate the degree of correlation. The final radiomics features were decided based on Spearman’s correlation coefficient >0.9 and significant p-value >0.01 after Holm-Bonferroni correction.

All statistical analyses were performed with RStudio software (version 1.1.456; RStudio, Inc., Boston, MA, United States) except for p-value of the overall model fit. Multivariable logistic regression models were developed integrating metastatic event and imaging features. The datasets were split into two random stratified cohorts: a training set (60%) and a test set (40%). Only the training set was used in the process to decide predictive and prognostic features. The 45 features were pre-selected, and then the bilateral correlation between these initial 45 features was evaluated with Spearman’s rank correlation coefficient in order to estimate potential redundancy between the features [16,18]. The threshold of testing correlation coefficient was higher than 0.9 [19]. To correct for multiple test comparisons, the Holm-Bonferroni correction method was applied for all p‘ values: the significance level was lower than a value p < p‘/m, where p‘ is 0.05 and m is the number of comparisons [20]. The calculated p-value was 0.01. The Spearman rank correlations for radiomics features are presented in Fig 1. The features from previous procedures were used to evaluate the relationship between them via the multiple backward stepwise elimination method based on Akaike’s Information Criterion (AIC) [9] in order to find the subset of features in the dataset resulting in the lowest prediction error. The determined features resulted from backward stepwise elimination and were assessed for overdispersion. The evaluation value was the ratio of residual deviance to residual degree of freedom (>1) and its p-value (>0.05). Subsequently, Friedman’s ANOVA test and odds-ratio were used to estimate their significances. They were also investigated for variable importance (varImp) and multicollinearity using variance inflation factor (VIF)—for which a value over the limit of four would indicate a problematic amount of collinearity. The logistic regression model was constructed again using the last decided features, and subsequently evaluated using the Hosmer-Lemeshow goodness of fit test. The p-value of the overall model fit was computed using MedCalc Statistical Software (version 18.10; MedCalc Software bvba, Ostend, Belgium).

### Model development and performance

The ratio of training set to test set was 60% to 40%. The training dataset (metastasis case: 27.45% and no metastasis: 72.55%) was used for training and validation and the test dataset (metastasis case: 25.00%% and no metastasis: 75.00%) was utilized only to assess its prediction. In the 10-fold cross-validation method, the training dataset was further split into a training set (90% of the data) and a validate set (10% of the data) randomly. One fold was reserved for validation, and the other nine folds were used to train the model. Subsequently, they were used to predict the target variable in the validating data. This process was repeated ten times, with the performance of each model in predicting the reserved set being tracked using a performance metric such as accuracy. The validated prediction model was applied to the test dataset that had not been used in the previous steps, and then the predicted values were compared to the actual values. Its outputs were evaluated in terms of area under curve (AUC), sensitivity, specificity, accuracy, and precision computed based on receiver-operating-characteristic (ROC) curves and confusion matrix.

### Predictive Tendency of Neoadjuvant Chemotherapy Prognosis and Metastasis

To analyze predictive tendency of neoadjuvant chemotherapy prognosis and metastasis, the radiomics features used for the radiomics model were investigated in four patient groups: Good-MetaFree (Good historic response and Metastasis Free), Poor-MetaFree (Poor historic response and Metastasis Free), Good-Meta (Good historic response and Metastasis), and Poor-Meta (Poor historic response and Metastasis) (Table 2).

## Results

### Feature selection and evaluations

The ratio of the patient’s data used for the training set and the test set was 60% to 40%. From 45 radiomics features, eight radiomics features were determined via Spearman’s rank correlation coefficient >0.9 and significant p-value >0.01 after the Holm-Bonferroni correction. These radiomics features were also decided after the logistic regression predictive model with backward stepwise elimination method was applied at 57.76 AIC. SUVmax and GLZM-SZLGE were eventually decided (Table 4).

**Table 4:** Radiomics features decided by Spearman’s rank correlation and backward stepwise elimination for use in multivariable regression analysis.

Classification of matrix	Features selected by Spearman’s correlation	Features selected by(Spearman’s correlation+backward stepwise elimination)
Conventional Indices	SUVmax MTV	SUVmax
Indices based on intensity histogram	Skewness	
Indices based on shape	Sphericity	
GLCM based on gray level co-occurrence matrix	Correlation	
NGLDM based on gray level neighborhood matrix	Contrast Busyness	
GLRLM based on gray level homogeneous run size matrix	GLNU	
GLZLM based on gray level homogeneous zone size matrix	SZLGE	SZLGE

SUVmax and GLZM-SZLGE were used for the multivariable logistic regression model and then evaluated using ANOVA test, odds ratio, varImp, and VIF (Table 5). SUVmax and GLZM-SZLGE showed less than 0.05 p-value in ANOVA test, higher than 1.0 Odds ratio, and their VIF values were higher than 4.0. The varImp test value of GLZM-SZLGE was higher than that of SUVmax. For overdispersion check, the ratio of residual deviance to residual degree of freedom was 1.078, and p-value was 0.30. The p-value of Hosmer-Lemeshow goodness of fit test was 0.5725. As a result, SUVmax and GLSZM-SZLGE were confirmed for the logistic regression model.

**Table 5:** Evaluation results of two radiomics features for use in multivariable regression analysis. The determined radiomic features were evaluated using ANOVA test, Odds ratio, varImp, and VIF. The results show that they are valid.

Classification of matrix	Index	ANOVA test	Odds Ratio (95% CI)	varImp	VIF
Conventional Indices	SUVmax	0.047	4.64 (1.03 to 20.97)	1.99	3.98
GLZLM	SZLGE	0.009	5.34 (1.38 to 20.73)	2.42	3.98

### Model development and performance

The trained and validated multivariable logistic model based on probability of endpoint P = 1/ (1+exp (-Z)) was

**Fig 2:**
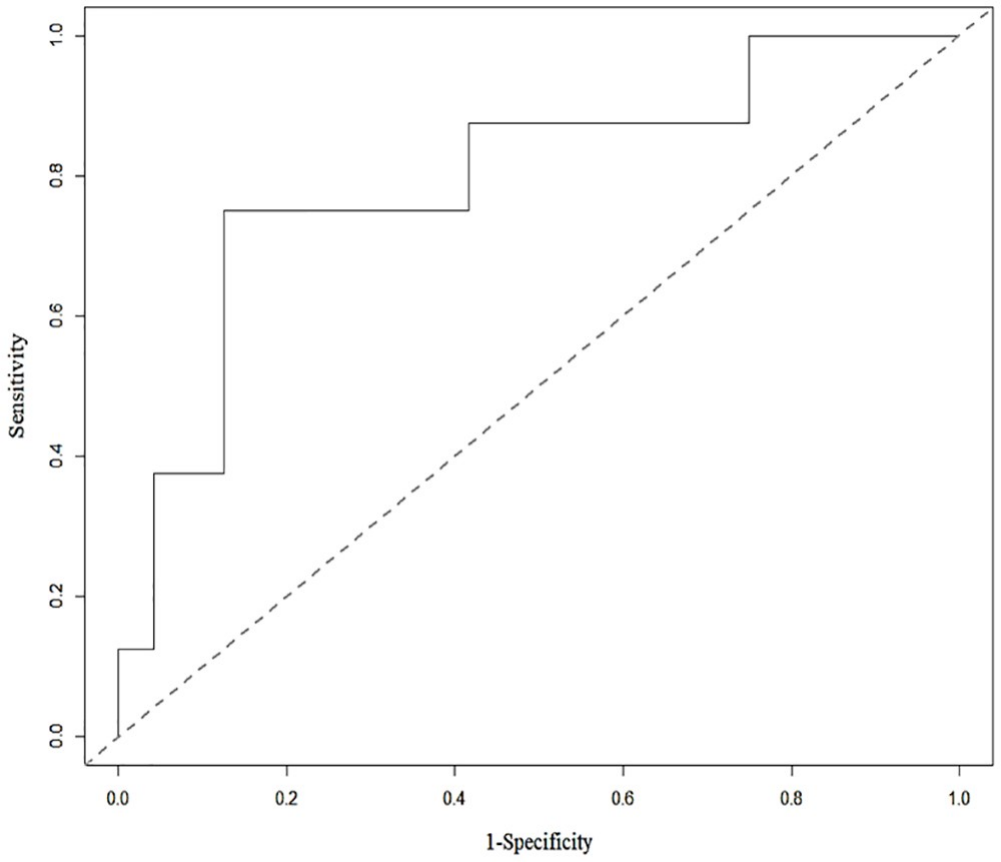
ROC curve for two radiomics features tested using the test dataset: SUVmax + GLZLM_SZLGE. The value shows that the classification based on texture analysis has good predictive value, as the area under the ROC curve is 0.80.

Z=−1.23+1.53*SUVmax+1.68*GLZLM−SZLGE

with significant p-values (SUVmax: 0.0462 and GLZLM-SZLGE: 0.0154). The developed predictive model was estimated using a test dataset that had not been used in previous procedures. Its AUC (Fig 2), accuracy, sensitivity, and specificity were 0.8, 0.88, 0.63, and 0.88 (Table 6).

**Table 6:** Estimation results of the predictive multivariable logistic model using the test dataset. AUV, accuracy, and specificity are good values while sensitivity is a fair value.

Predictive multivariable logistic model	probability of metastasis, $P=\frac{1}{\left(1+e^{-z}\right)}$			
	Z = -1.23 + 1.53*SUVmax + 1.68*GLZLM_SZLGE			
Estimation item	AUC	Accuracy	Sensitivity	Specificity
Results	0.80	0.81	0.63	0.88

### Predictive Tendency of Neoadjuvant Chemotherapy Prognosis and Metastasis

**Fig 3:**
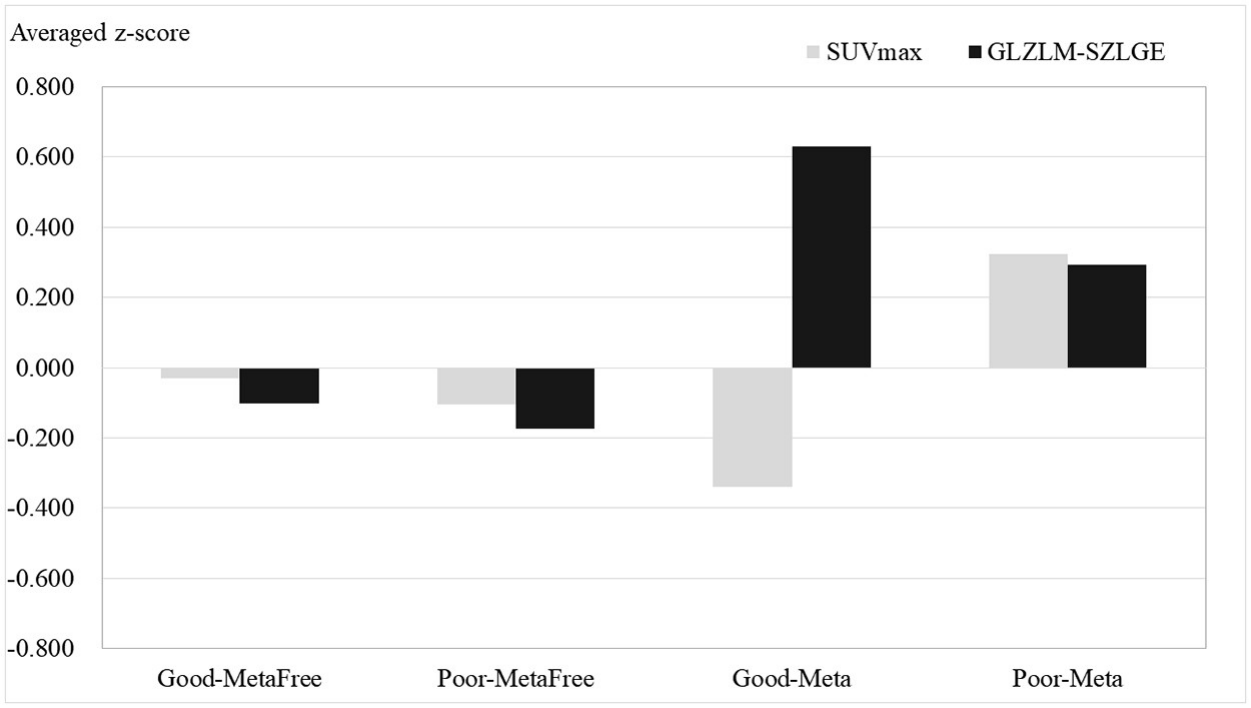
Histogram of the averaged z-scores of SUVmax and GLZLM-SZLGE for the historic response prognosis and metastasis. Regardless of historic response outcome, GLZLM-SZLGE presents positive value for the metastasis patient group, while it shows negative value for the metastasis free patient group. SUVmax has no related tendency.

The predictive tendency of historic response prognosis and metastasis was investigated using SUVmax and GLZLM-SZLGE (Fig 3). Both features were negative in the Good-MetaFree group and Poor-MetaFree group, while they were positive in the Poor-Meta group. In the Good-Meta group, SUVmax was negative, but GLZLM-SZLGE was positive.

## Discussion

In this study, a multivariable logistic regression imaging model was developed for the prediction of future metastases development at the point of osteosarcoma diagnosis. The model consists of two radiomics features extracted from ^18^F-FDGPET. The selection of radiomics features comprises several suggested steps that remove the redundancy between radiomics features and its overfitting. In order to strengthen the clinical impact of the model, the images used were acquired and reconstructed under routine clinical conditions. It is expected that the methodology used in this study could be generalized to other types of cancer and tumor outcomes.

The association of selected radiomics features during processing with metastases development in osteosarcoma is shown in Tables 3 and 4. The partial volume effect was not an issue in this study because all tumor volumes were higher than 8.39 cm^3^ (matrix size: 4.063 mm × 4.063 mm and slice thickness: 3.03 mm). Eight independent radiomics features resulted from Spearman rank correlation based on all matrix classifications. The results indicate that SUVmax was correlated to other SUVs (SUVmean and SUVpeak) and twenty-seven other texture radiomics features were associated with homogeneity, uniformity, inertia, randomness, dissimilarity, run-length matrix, and zone-size matrix of gray level. Further, it was related to global radiomics features and higher radiomics features, but it was not correlated to any feature from NGLCM. On the other hand, MTV was interrelated with six features related to TLG, shape, edge, and similarity of intensity values, and it was related to coarseness from NGLDM. Skewness (asymmetry of the histogram) based on histogram was correlated to its kurtosis (flatness of the histogram). The remaining six radiomics features (Shape-Sphericity, GLCM-Correlation, GLRLM-GLNU, NGLCM-Contrast, NGLCM-Busyness, and GLZLM-SZLGE) were not dependent on any other features. These features were investigated via varImp and the backward stepwise elimination method based on AIC.

SUVmax and GLZM-SZLGE were finally decided as predictors for the multivariable logistic regression model, and were verified based on the results of Friedman’s ANOVA test (p-value < 0.05), odds-ratio (> 1.0), multicollinearity (VIF < 4.0), and varImp. The developed multivariable logistic model with these predictors was suggested as a good fit model without overdispersion after a Hosmer-Lemeshow goodness of fit test and overdispersion evaluation. After the finalized multivariable logistic model was trained and validated using the 10-folds cross-validation method, it was applied to the test dataset. For this predictive model, good evaluation results were obtained: AUC, accuracy, and specificity were 0.8, 0.81, and 0.88, respectively. The sensitivity (0.63) was lower than other values, although it was fair. This occurred because of the insufficient number of metastasis cases—only eight. As a result, the multivariable logistic model built via linear combination of two verified radiomics features (SUVmax and GLZM-SZLGE) was confirmed with high predictive potential for metastases in osteosarcoma.

In this study, SUVmax and GLZM-SZLGE were finalized as the predictors for the radiomics model. SUVmax reflects the tumor aggressiveness and is an independent prognostic factor [4]. As a result, the positive correlation of SUVmax with metastases confirms that high-uptake in ^18^FDG-PET can play an important role in the characterization of aggressive tumors. These results are congruent with published papers that state that it could be a clinical indicator of sarcoma [20–24]. It was also used as an indicator of histopathologic response after neoadjuvant chemotherapy in extremity osteosarcoma in our previous report [25]. The other predictor, GLZM-SZLGE, increases when the short runs with low gray value are dominant. The positive correlation of GLZM-SZLGE indicates that many short runs of low gray value dominate in the fine-grained texture of the tumor. Consequently, higher SUVmax and GLZM-SZLGE result in higher possibility of tumor metastasis. They could imply tumor biological phenomenon based on the Warburg effect [26–28]. Tumor cells at sites close to blood vessels are relatively sufficiently oxygenated, whereas those located further away are hypoxic. This phenomenon is important for secondary tumor events as hypoxia can independently induce the epithelial–mesenchymal transition (EMT), a crucial step in cancer progression and metastasis via a number of mechanisms such as HIF-1α expression in osteosarcoma and osteosarcoma cells [29–31]. Considering our results, SUVmax can be recognized as the most proliferative region by tumor cells residing closer to blood vessels. In contrast, GLAM-SZLAG can be considered a hypoxic region by those residing further from it. It can be concluded that SUVmax and GLAM-SZLAG related with metastasis could provide information about tumor latent biology. Additionally, these two features could differentiate between two AJCC stages (IIA and IIB) with less than 0.05 p-value when the correlation between those stages and the AJCC stage commonly used for predicting outcome was considered (S1 Table).

Fig 3 shows the predictive tendency of historic response prognosis and metastasis. Whether the historic response outcome was good or poor, the averaged z-score of GLZLM-SZLGE was positive in the metastasis patient group. In contrast, it was negative in the metastasis free patient group. SUVmax did not show the relative tendency. Our results highlighted the Good-Meta group, which indicated good historic response of neoadjuvant chemotherapy but the primary tumor was metastasized in the endpoint. The conventional value, SUVmax, could not drive a high possibility of metastasis because it was too low. However, the radiomics feature, GLZLM-SZLGE, could predict the possibility of metastasis. We demonstrated that the texture-based radiomics model could supplement clinical prognostic factors for optimal prediction. However, multinomial logistic regression for the histological response and metastasis could not be achieved owing to insufficient patient cohorts.

In this study, the potential association between radiomics features and known metastatic colonization was presented, but biological information from the same patient was not used. However, this is required in order to assess the association and predictive power of the radiomics features for tumor cell information acquired from a patient. In addition, a larger patient cohort is essential in order to develop a more powerful model. Further, in order to facilitate clinical implementation of a texture-based decision-support system, harmonization and standardization on data and methods are also obviously required.

## Conclusion

Texture biomarkers derived from ^18^F-FDG images have been attracting attention as promising tools for characterizing spatial heterogeneity in order to predict tumor consequences at an early stage in humans. Verified selection of texture extraction and multivariable logistic modeling approaches have been proposed for the development of tumor outcome prediction models from a number of radiomics features in this study. The findings show that ^18^F-FDG image features could perform as strong prognostic and predictive factors of metastasis in osteosarcoma and could provide observations about their inherent biology. The findings also indicate the priority of optimizing radiomics features to build up their predictive value and to determine the relation between features and biology. This model achieved good prediction performance evaluations in cross-validation, and its predictive properties were confirmed using an independent test dataset. However, further validation is required for stronger and more robust predictive properties. The established methodology can be applied to other tumors and could ultimately induce progress in treatment personalization and patient survival.

## Supporting information

S1 Table Average values of SUVmax and GLZLM_SZLGE and their p-value results from two-sample t-test in IIA and IIB. (DOCX)
