# Supplementary material for: Metastasis risk prediction model in osteosarcoma using metabolic imaging phenotypes: A multivariable radiomics model
Source: PLoS One. 2019 Nov 25;14(11):e0225242. doi: 10.1371/journal.pone.0225242 (PMC6876771; doi:10.1371/journal.pone.0225242)
Supplement: S1 Table — (DOCX) [file pone.0225242.s001.docx]

**Supplementary Table**

**Legend to Supplementary Table**

**Supplementary Table 1:**

**Average values of SUVmax and GLZLM_SZLGE and their p-value results from two-sample t-test in IIA and IIB**

The difference between IIA and IIB in SUVmax and GLZLM-SZLGE was compared using two-sample t-test.Tthese two features could differentiate between two AJCC stages (IIA and IIB) with less than 0.05 p-value when the correlation between those stages and the AJCC stage commonly used for predicting outcome was considered.

**Supplementary Table 1:**

| AJCC stage | SUVmax | GLZLM_SZLGE |
| --- | --- | --- |
| IIA | 6.786 ± 2.515 | 0.005 ± 0.001 |
| IIB | 10.2927 ± 5.001 | 0.004 ± 0.001 |
| p-value | 0.0004 | 0.013 |
